# Molecular Targets for Coevolutionary Interactions Between Pacific Oyster Larvae and Their Sympatric *Vibrios*

**DOI:** 10.3389/fmicb.2019.02067

**Published:** 2019-09-06

**Authors:** K. Mathias Wegner, Damien Piel, Maxime Bruto, Uwe John, Zhijuan Mao, Marianne Alunno-Bruscia, Bruno Petton, Frédérique Le Roux

**Affiliations:** ^1^Coastal Ecology, Waddensea Station Sylt, Alfred Wegener Institut - Helmholtz-Zentrum für Polar- und Meeresforschung, List, Germany; ^2^Unité Physiologie Fonctionnelle des Organismes Marins, Ifremer, Plouzané, France; ^3^Integrative Biology of Marine Models, UPMC Paris 06, CNRS, UMR 8227, Sorbonne Universités, Station Biologique de Roscoff, Roscoff, France; ^4^Ecolgical Chemistry, Alfred Wegener Institut - Helmholtz-Zentrum für Polar- und Meeresforschung, Bremerhaven, Germany; ^5^Helmholtz Institute for Functional Marine Biodiversity (HIFMB), Oldenburg, Germany; ^6^Biological and Environmental College, Zhejiang Wanli University, Ningbo, China; ^7^LEMAR UMR 6539, Ifremer, Argenton-en-Landunvez, France

**Keywords:** virulence, host-pathogen interaction, local adaptation, biological invasion, emerging disease, Wadden Sea

## Abstract

Bacteria of the *Vibrio* genus are the most predominant infectious agents threatening marine wildlife and aquaculture. Due to the large genetic diversity of these pathogens, the molecular determinants of *Vibrio* virulence are only poorly understood. Furthermore, studies tend to ignore co-evolutionary interactions between different host populations and their locally encountered *Vibrio* communities. Here, we explore the molecular targets of such co-evolutionary interactions by analyzing the genomes of nine *Vibrio* strains from the *Splendidus-*clade showing opposite virulence patterns towards two populations of Pacific oysters introduced into European Wadden Sea. By contrasting *Vibrio* phylogeny to their host specific virulence patterns, we could identify two core genome genes (OG1907 and OG 3159) that determine the genotype by genotype (G × G) interactions between oyster larvae and their sympatric *Vibrio* communities. Both genes show positive selection between locations targeting only few amino acid positions. Deletion of each gene led to a loss of the host specific virulence patterns while complementation with OG3159 alleles from both locations could recreate the wild type phenotypes matching the origin of the allele. This indicates that both genes can act as a genetic switch for *Vibrio*-oyster coevolution demonstrating that local adaptation in distinct *Vibrio* lineages can rely on only few genes independent of larger pathogenicity islands or plasmids.

## Introduction

*Vibrionaceae* (thereafter named vibrios) are among the most common and wide spread infectious disease agents of marine wildlife (Hada et al., 1984; Rosenberg and Ben-Haim, 2002; Austin and Zhang, 2006; Austin and Austin, 2007; Perez-Cataluna et al., 2016) and cause substantial damage to the aquaculture industry, especially to that of bivalves (Paillard et al., 2004; Romalde et al., 2014; Travers et al., 2015; Dubert et al., 2017). Vibrios are genetically (Thompson et al., 2005) and ecologically (Takemura et al., 2014) highly diverse and their niche flexibility has partly been attributed to their ability to exchange genetic material by horizontal gene transfer (Le Roux and Blokesch, 2018). The high genetic diversity alone complicates our understanding of virulence mechanisms, especially since multiple factors can act in concert (Le Roux and Blokesch, 2018). In this context, the repeated mass mortalities of Pacific oysters *Crassostrea gigas* associated to diverse populations of vibrios represent an excellent case study of *Vibrio*-related disease in natural and economically important systems (Le Roux et al., 2016).

Only a few virulence genes of vibrios infecting oysters have been functionally characterized. These include secreted metalloproteases (Le Roux et al., 2007; Binesse et al., 2008; Hasegawa et al., 2008; Labreuche et al., 2010), vesicular serine protease (Vanhove et al., 2015), outer membrane protein U (Duperthuy et al., 2010, 2011), resistance genes against oxidative stress or heavy metals, (*sodA*, *copA*; Vanhove et al., 2016), toxins (MARTX, Bruto et al., 2018) or regulators (*varS*, Goudenege et al., 2014). Others lack functional annotation despite being widespread throughout the *Vibrio* phylogeny (Lemire et al., 2014; Bruto et al., 2018), and especially the discovery of the functional importance of these uncharacterized genes highlights the need to employ a consequent combination of comparative genomics with functional genetics to grasp the full variety of virulence factors in *Vibrio*-oyster interactions.

To date, most studies have focused on vibrios associated with disease outbreaks in aquaculture favoring isolates from diseased animals that probably show higher virulence (Sundberg et al., 2016). Aquaculture practices (e.g., high density of farmed animal, use of antibiotics, and transfer of animals) radically alter the ecology of both host and pathogens (Pulkkinen et al., 2010). In that context it can be assumed that bacterial disease emerged after rapid spread of selectively favored virulence factors by horizontal transfer of large genomic islands or plasmids (Bruto et al., 2017; Le Roux and Blokesch, 2018).

In natural populations, the extended contact between host and parasite/pathogen over several generations can lead to an arms race like host parasite interaction, characterized by fast reciprocal evolutionary changes of both parties, i.e., coevolution (Buckling and Rainey, 2002; Decaestecker et al., 2007; Schulte et al., 2010; Eizaguirre et al., 2012). Within populations this fast reciprocal change can lead to strong genotype by genotype (GxG) interactions and fluctuating selection on host and parasite genotypes (Decaestecker et al., 2007) that maintains genetic diversity (Berenos et al., 2011). On larger geographic scales, asynchrony of coevolution between populations can lead to between-population differentiation of both parasites and hosts in geographic mosaics of coevolution (Thompson et al., 2005). Host-parasite coevolution thus leads to local adaptation of host and parasite genotypes (Kawecki and Ebert, 2004) that has been observed in several marine host-parasite pairs (Sotka, 2005). Furthermore, virulence-transmission trade-offs can lead to selection of lower virulence to optimize transmission and maximize basic reproductive rate R_0_ (Acevedo et al., 2019). Identification of virulence factors in *Vibrio*-oyster interactions has so far ignored such coevolutionary interactions, despite of the ecological and evolutionary importance of host genetic background as one decisive determinant of disease outcome (Le Roux et al., 2016).

**FIGURE 1:**
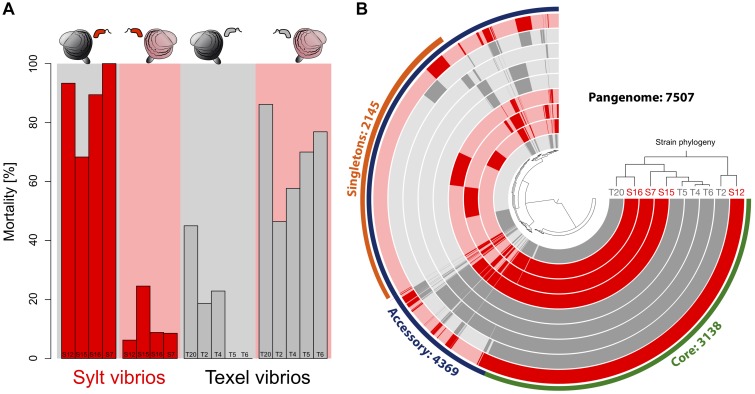
**(A)** Specific virulence patterns of the *Vibrio* strains used for sequencing. Strains were isolated from Sylt and Texel oysters and tested on sympatric and allopatric oyster larvae. Red bars represent Sylt strains (S12, S15, S16, S7) and gray bars represent Texel strains (T20, T2, T4, T5, T6). A red background shows infection on Sylt oysters and a gray background infections on Texel oysters. **(B)** Pangenome of the Sylt and Texel *Vibrio* strains. Strains are ordered according to their phylogenetic tree. Strains from Texel are colored in gray while strains from Sylt are colored in red. A circular track shows the presence/absence of gene families. Core gene families that were present in all genomes are marked in green, and accessory gene families that were present in at least one strain are marked in blue. Within the accessory genome singleton genes are shown in orange. Gene families are organized in clusters (distance method = Euclidean; clustering method = Ward).

Here, we now exploit such coevolutionary interactions to identify virulence factors of vibrios from the *Splendidus*-clade that cause host-specific mortality patterns in natural beds of Pacific oysters in the North Sea. The *Splendidus*-clade is a diverse and species-rich group of vibrios that ancestrally carries several virulence factors (Bruto et al., 2018). Infection of larvae from two distinct oyster populations with sympatric or allopatric vibrios from the *Splendidus*-clade already showed that patterns of local adaptation can arise quickly after oysters have been introduced (Wendling and Wegner, 2015). In detail, *Vibrio* spp. from either location (Texel in the Southern Wadden Sea and Sylt in the Northern Wadden Sea) were much less harmful to sympatric oyster larvae from the same location than to allopatric isolates, i.e., high mortality on Sylt larvae was coupled to low mortality on Texel larvae and vice versa (Wendling and Wegner, 2015). These oyster populations represent two distinct invasion events and are genetically differentiated (Moehler et al., 2011). Crosses between Sylt and Texel oysters revealed a dominant inheritance of host resistance in F1 larvae of mixed genetic background suggesting that specificity between vibrios and oysters relies on few loci in the oyster host (Wendling and Wegner, 2015). If host resistance relies on few loci, it is likely that also the number of interacting genetic components on the pathogen side is small helping the identification of virulence factors. Furthermore, since directly opposite mortality patterns on sympatric and allopatric oysters were observed in several distinct *Vibrio* species found in both locations, we could single out pairs of phylogenetically closely related strains from both locations for our analyses (Figure 1). This choice of strains will thus generate a strong contrast between genome-wide phylogeny (i.e., the species tree) and gene specific phylogenies associated with the contrasted phenotypes (i.e., the phenotype tree). Within nine phenotypically and phylogenetically contrasted *Vibrio* genomes we could identify two widespread yet uncharacterized genes from the core genome that determined interactions between oyster larvae and their sympatric *Vibrio* communities. Furthermore, expression of alleles from both locations demonstrated the functional involvement of these genes, because phenotypes matching the origin of the allele in a knock out mutant could be recreated. By estimating synonymous and non-synonymous substitution rates (i.e., dN/dS) within and between locations we could further identify the specific amino acid positions responding to selection and leading to the genotype by genotype (G × G) interactions. This highlights the use of coevolution in natural populations to complete our understanding of the genetic architecture and molecular pathways of virulence phenotypes in *Vibrio*-oyster interactions.

## Materials and Methods

### Strain Selection

The vast majority *Vibrio* strains previously characterized for specific virulence patterns in oyster larvae along a geographic gradient belonged to the *Splendidus*-clade (70 out of 76, Wendling and Wegner, 2015). Therefore, we focused on this group and chose strains that came from the same location as the oyster brood stock (i.e., Sylt and Texel) to ensure the possibility for local coevolutionary interactions. Additionally, the chosen strains had to cause significantly less mortality on their sympatric host than on their allopatric one. This included the majority of strains isolated on Sylt (10 out of 18) and on Texel (6 out of 10). To maximize and replicate the contrast between phylogeny and virulence phenotype we then identified nine strains (S7, S12, S15, S16 isolated on Sylt and T2, T4, T5, T6, and T20 isolated on Texel) from different taxonomic species for genome sequencing based on previous MLST genotyping (Wendling and Wegner, 2015).

### Genome Sequencing

High molecular weight DNA was extracted from overnight cultures using the Bacterial DNA kit (Sigma-Aldrich, Munich, Germany) following the manufacturers instructions. After inspection of DNA amount and integrity paired-end library preparation and sequencing was performed using the Illumina NGS platform and methodology. Approximately 5 μg of DNA was used for PE library preparation according to the manufacturer’s instructions (Illumina PE sample preparation kit). The libraries were sequenced on a HiSeq2000 in 100 bp PE mode. Reads were extracted in FastQ format using CASAVA v1.8.2 (supported by Illumina).

### Bioinformatic Analyses

Raw sequencing reads were quality checked and trimmed of adapter sequences before they were assembled using the *de novo* assembly algorithm implemented in the CLC genomics workbench (CLC, Arhus, Denmark). High quality contigs are available under the accession numbers at NCBI (T20: VFEB00000000; T6: VFEC00000000; T5: VFED00000000; T4: VFEE00000000; T2: VFEF00000000; S7: VFEG00000000; S15: VFEH00000000; S12: VFEI00000000; S16: VFEJ00000000) and were annotated using the automated RAST pipeline at http://rast.nmpdr.org. A genome wide phylogeny of the strains sequenced here was based on a distance matrix of pairwise distances between genomes computed by the software *andi* (Haubold et al., 2015). The distances were clustered using the neighbor joining algorithm implemented in the *neighbor* routine of the PHYLIP software suite available at http://evolution.genetics.washington.edu/phylip.html.

Based on the gene annotations we constructed the accessory and core genome. We used OrthoMCL to identify orthologous groups from the genes predicted by the RAST annotation (Li et al., 2003). OrthoMCL uses a similarity matrix from an all-against-all protein BLAST (e cut-off = 10^–5^) with subsequent Markov clustering to identify groups of orthologous genes and recent within species paralogs. The graphical representation of the pangenome encompassing all gene families was drawn with Anvio (Delmont and Eren, 2018). In order to study the accessory genome, we searched for gene families that formed gene pools that were exclusively shared by all strains from the same location. For the core genome analysis, we concentrated on 1:1 orthologs, i.e., genes with no paralogs within any strain. For this set of 1:1 orthologs we then calculated a genetic pairwise distance matrix based on the rate of non-synonmous substitutions (dN) by translating the protein alignment back to nucleotides using transAlign (Bininda-Emonds, 2005) and applying the *kaks* function of the *ade4* R package. Each resulting distance matrix was then correlated to the distance matrix from the whole genome sequence calculated by *andi* (see above) to obtain the phylogenetic correlation for each orthologous group using mantel tests. We then correlated the genetic distance matrix of each orthologous group to the phenotypic distance matrix obtained from the virulence profiles. Also here mantel tests were performed and topologies of unrooted trees were checked for significant matrix correlations.

Functional annotation of candidate genes was obtained by identifying conserved protein domains in pfam and NCBI-CDD using *motif* available at https://www.genome.jp/tools/motif/ with a cutoff e-04. Localization in the cell was predicted using PSORTb available at https://www.psort.org/psortb/ with standard options. Transmembrane structure and signal peptide presence was determined via PHOBIUS (Kall et al., 2007).

Patterns of adaptive evolution were estimated by counting synonymous and non-synonymous substitutions. We grouped those in substitution that were fixed within locations (i.e., divergence substitutions D_n_ and D_s_) and polymorphic substitutions (P_n_ and P_s_) and calculated the Direction of Selection [DoS = D_n__/_(D_n_+D_s_)−P_n__/_(P_n_+P_s_)] as a less biased test statistic for the McDonald-Kreitman contingency table (Stoletzki and Eyre-Walker, 2011). Position specific signatures of selection were determined by ratios of non-synonymous to synonymous substitutions rates (dN/dS) along the protein sequence. We used Bayesian Markov chain Monte Carlo procedures implemented in omegaMap (Wilson and McVean, 2006) to calculate strength of selection by combining two independent runs of 100000 iterations and a thinning interval of 10 that was based on three random input orders of sequences. We used weak but proper inverse priors and determined dN/dS in a sliding window block size of 3 codons along the sequence. To differentiate diversifying selection within locations from positive selection between locations we ran this analysis with all sequences from Sylt and Texel as well as with sequences from each location separately.

### Molecular Microbiology

Strains and plasmids used or constructed in the present study are described in Supplementary Table S2. *Vibrio* isolates were grown at 20°C in Luria-Bertani (LB) or LB-agar (LBA) + 0.5 M NaCl. *Escherichia coli* strains were grown at 37°C in LB or on LBA. Chloramphenicol (Cm, 5 or 25 μg.ml^–1^ for vibrios and *E. coli*, respectively), thymidine (0.3 mM) and diaminopimelate (0.3 mM) were added as supplements when necessary. Induction of the P_BAD_ promoter was achieved by the addition of 0.02% L-arabinose to the growth media, and conversely, was repressed by the addition of 1% D-glucose. Deletion of selected regions or genes was performed by allelic exchange using the pSW7848T suicide plasmid (Val et al., 2012). To this end, two 500 bp fragments flanking the target gene were amplified and assembled by PCR (see primer details in Supplementary Table S1), cloned into pSW7848T as previously described (Lemire et al., 2014) and transferred by conjugation from *E. coli* donor and the *Vibrio* recipient strains. Subsequently, the first and second recombination events leading to pSW7848T integration and elimination were selected on Cm/glucose and arabinose containing media, respectively. For complementation experiments, the two alleles of OG3159 or *gfp* as a control were cloned into the Apa1/Xho1 sites of the pMRB plasmid resulting in a constitutive expression from a P_LAC_ promoter (Le Roux et al., 2011; Lemire et al., 2014). Conjugation between *E. coli* and *Vibrio* strains were performed at 30°C as described previously (Le Roux et al., 2007).

### Experimental Infections

Virulence of wild type or mutant derivatives was tested on 3 day old oyster larvae by challenging 10–20 larvae with a concentration of 10^7^ cells ml^–1^ in triple replicates. Dead larvae were counted after 24 h of exposure and mortality rates were calculated as the fraction of dead larvae of the total larvae, which were counted after the end of the experiment by killing all larvae. All mutants (two clones per gene) were tested on larvae from Texel and Sylt produced under standardized conditions (Helm and Bourne, 2004). After showing that the knock-outs worked in both larvae sources, we only tested complementation mutants on Sylt larvae as a proof of principle for the control of virulence patterns by the location specific alleles. Mortality proportions were analyzed by binomial generalized linear models (GLM) taking the number of dead larvae vs. the number of living larvae on day 2 of the experiment as a function of *Vibrio* strain (WT, knockout) and host origin (Sylt, Texel). Differences between groups of significant interactions and different factor levels were then tested by Tukey’s *post hoc* test implemented in the R package *multcomp* (Hothorn et al., 2008).

## Results

### Taxonomic Assignment Based on Genome Sequence Analyses

**FIGURE 2:**
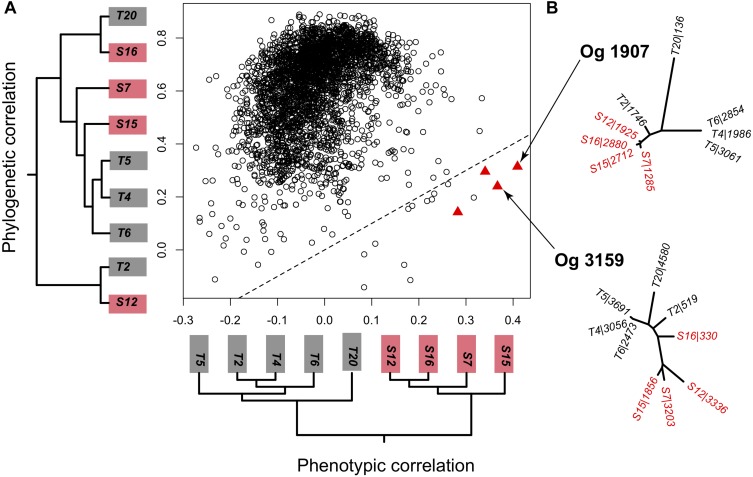
**(A)** Correlation of each orthologous single-copy gene present in every strain to the genomewide phylogeny shown on the *y* axis (phylogenetic correlation) and to the phenotype tree reflecting virulence on the *x* axis (phenotypic correlation). Out of the 3089 1 on 1 orthologous groups (OG) only 4 showed a significant correlation to the phenotype tree that was higher than the phylogenetic correlation and only two of those produced a phylogenetic tree topography that was compatible to a split into two local groups. **(B)** Unrooted phylogenies of the orthologous genes OG1907 and OG3159 that significantly correlated to the phenotype matrix. Trees were based on a dN distance matrix. Labels show the strain identity and the number of the corresponding predicted protein-encoding gene.

To explore molecular targets for local adaptation, we selected *Vibrio* strains from the *Splendidus*-clade isolated on Texel (T2, T4, T5, T6, T20) or Sylt (S7, S12, S15, S16) that were representative of the prevalent patterns of opposite mortality patterns on sympatric and allopatric oysters (Figure 1A). The *de novo* assembly of the nine genomes resulted in ∼99% coverage with genome size ranging from 4.5 to 5.6 Mb in length corresponding to 4013 genes and 4894 predicted genes for T2 and S7, respectively (Table 1). The genome-wide phylogeny (Figure 1B) confirmed the phylogenetic placement within the *Splendidus* clade of the previous classification by Multi Locus Sequencing Tag (MLST) analyses (Wendling and Wegner, 2015) for the majority of the strains. Strain T4, which was paired to S7 based on MLST, was, however, more closely related to T5 and T6 based on the whole genome phylogeny (Figure 1B). While this reduced the phylogenetic variability within the Texel strains, the contrast between phylogeny and virulence phenotypes (Figure 2) was still sufficiently large to identify the targeted virulence genes.

**TABLE 1:** Summary statistics of assembly and annotation for the sequenced *Vibrio* genomes.

**Statistic**	**S7**	**S12**	**S15**	**S16**	**T2**	**T4**	**T5**	**T6**	**T20**
# of reads (millions)	20.5	18.7	18.0	22.6	20.5	21.8	13.9	17.8	17.3
# of nucleotides (millions)	1975.3	1824.9	1750.1	2225.4	2012.5	2156.0	1378.7	1752.2	1699.8
# of contigs	257	155	450	400	192	318	235	114	259
N50	217’830	205’529	146’600	133’110	191’265	150’386	146’732	229’145	122’250
N75	109’863	95’225	78’430	76’994	82’060	80’151	95’051	120’531	215’231
Largest contig	669’531	341’281	591,072	336’801	511’873	612’686	612’755	755’092	468’870
Average sequencing depth	355	402	325	423	445	414	266	360	314
Estimated genome size	5’559’262	4’543’720	5’391’904	5’258’494	4’520’555	5’208’681	5’182’738	4’867’815	5412’861
Genome coverage	≈99%	≈99%	≈99%	≈99%	≈99%	≈99%	≈99%	≈99%	≈99%
GC content	44.0	43.9	43.6	44.3	43.8	43.5	43.5	43.6	44.1
Predicted genes	4894	4022	4738	4576	4013	4542	4507	4253	4706

### Two Core Genes Endorse Opposite Virulence on Sympatric and Allopatric Oysters

Virulence of *Vibrio* isolates toward allopatric hosts either suggests that: (i) isolates from the same location share a common and specific gene pool; (ii) adaptive alleles of core genes involved in virulence segregate between the different geographical areas. To disentangle these hypotheses, we first explored the gene content and distribution of the flexible genome. The pan-genome of all nine strains consists of 7507 gene families encompassing 3138 core-genes (present in all 9 strains) and 4369 accessory gene families (present in 1 to 8 strains) (Figure 1B). None of the accessory genes was specifically found in all isolates from Texel and absent in all Sylt strains. On the other hand, six genes were only found in Sylt strains and absent in Texel strains. These genes encode a hypothetical protein, a putative orphan protein, a K + transporter, a *LysR* transcriptional regulator, and a small subunit of a D-amino acid dehydrogenase and are preceded upstream by an *integrase* gene suggesting that this cluster represents a local phage-mediated gene transfer. Since we could not observe a similar cluster within the Texel strains nor any Texel-specific genes, the content of the accessory genome, *i.e.*, a specific gene pool shared by all strains from the same geographical area, can probably not explain the contrasted host specific phenotypes.

We therefore focused on the core genome to identify orthologous core-gene groups (OG) where higher sequence diversity between than within geographical areas could explain the specific virulence patterns. Out of the 3138 OGs with representatives in each strain, 3089 OGs had only a single representative per strains (i.e., were 1 on 1 orthologs). The gene-based phylogeny reflected the species-based phylogeny for the majority of orthologous groups (2747 OGs with Mantel tests *p*-value < 0.05, Figure 2A). In contrast, only four genes correlated significantly with the virulence phenotype tree (Figure 2A, red triangles). For two out of these four orthologous groups, tree topologies did not match the expected split between locations dictated by the virulence phenotypes. This indicates that a few large pairwise distances drove the correlation with virulence but not the tree topology. The other two OGs (OG1907 and OG3159) resembled the topology predicted from the phenotype tree (Figure 2B) and thus represent likely candidate genes underlying local virulence patterns.

**FIGURE 3:**
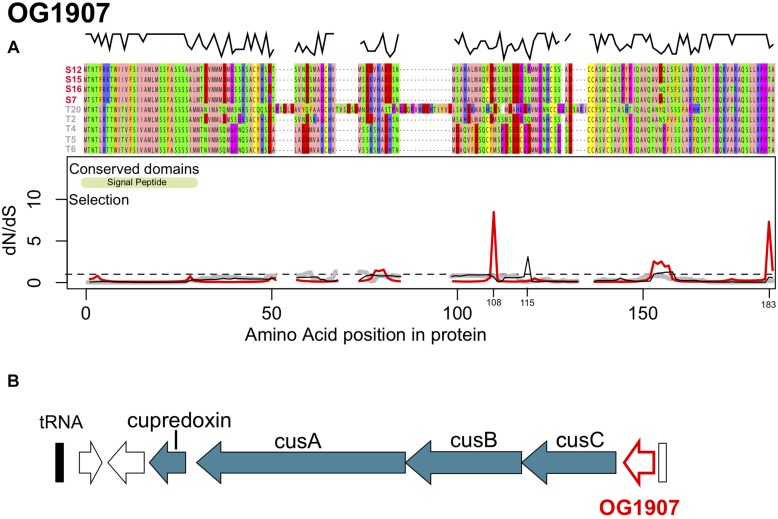
**(A)** Protein alignment, conserved domains and selection along the protein sequence of OG1907. The alignment was color coded according to the Zappo color scheme according to their physicochemical properties. The black line above the alignment displays sequence similarity within the alignment. Synonymous to non-synonymous substitution rates (dN/dS) along the sequence are shown for Sylt sequences (red), Texel sequences (gray) and all sequences (Sylt + Texel, black), thus showing contributions of within vs. between location responses to selection. The dashed line shows neutral evolution while peaks above this line indicate positive selection. **(B)** Genomic context of OG1907 showing its tight link to the *cusABC* copper transporter.

The first orthologous group OG1907 encodes a 154–184 amino acid long protein with a theoretical molecular mass of 16.5 kDa and isoelectric point of 5.15 (Figure 3A). OG1907 in strain T20 showed reduced similarity to the other sequences due to several insertions elongating the protein alignment to184 amino acids (Figure 3A). Annotation using pSORTb and SigmaP predicted an extracellular localization and the presence of a signal peptide at position 1–29 (Figure 3A). Only Sylt sequences were predicted to contain a Glycosyl Hydrolase 3 domain (PF00933, *e* = 6.2e^–04^) from position 21 to 96, which was not predicted for any of the Texel sequences. In all strain genomes, OG1907 is localized upstream of genes encoding *cusABC*, which are predicted to form a copper efflux pump (Pal et al., 2017; Figure 3B). The close association to *cusABC* together with its extracellular localization might therefore suggest that OG1907 encodes an extracellular protein involved in copper homeostasis by a mechanism that remains to be determined.

Overall, owing to the insertions in T20 and the high number of substitutions in T2 compared to the similar T4, T5, and T6 sequence similarity among strains was comparatively low along the sequence and only the terminal parts of the protein were reasonably conserved (Figure 3A). Low sequence similarity was, however, mainly found in sequences from Texel strains, while Sylt sequences were highly similiar. Accordingly, similarities within Texel strains (79 ± 15%) were significantly lower than within Sylt strains (97 ± 1.1%, *t* = −3.449, *p* = 0.004). Similarities between Sylt and Texel strains (76 ± 7.4%) were also significantly lower than within Sylt strains (*t* = −4.467, *p* < 0.001), but did not differ from within Texel diversity (*t* = 0.770, *p* = 0.721) indicating that differentiation between the locations was mainly caused by the highly similar alleles within Sylt strains whereas Texel strains accumulated alleles reflecting the whole diversity of the gene (Figure 3B). While differentiation between locations was reflected in a comparatively high number of non-synonymous fixed and polymorphic substitutions, especially synonymous polymorphic substitutions accumulated at a lower rate (Table 2), indicating only weak directional selection between locations. Accordingly, these substitution patterns resulted in a Direction of selection coefficient of DoS = −0.065 indicating rather neutral than adaptive evolution between locations. Whole gene estimates of selection can incorporate a mix of sites under purifying selection (dN/dS < 1) and sites under positive selection (dN/dS > 1) that cancel each other out in the overall analysis. This also applied to OG1907 where specific positions in the alignment showed signatures of strong positive selection with dN/dS ratios reaching as high as 8.48 for position 108 (Figure 3A, red line). Together with a second peak of selection in OG1907 at position 183 of the alignment (matching position 152 of the protein in T2) with a dN/dS ration of 7.303 these targets of selection were found in the data set only containing Sylt sequences, suggesting that local diversifying selection maintained polymorphism within this population despite of high overall sequence similarities in this group. To explain location specific virulence patterns, we needed to identify selection acting between locations rather than within. Therefore, we compared dN/dS ratios in the data set containing all sequences to the split data set containing sequences from each location separately. Amino acid position 115 showed such a between location target of selection with a dN/dS ratio of 3.113 for the complete data set (Figure 3A, black line), while purifying selection could be detected within the respective Sylt (dN/dS = 0.511) or Texel (dN/dS = 0.763) data sets.

**TABLE 2:** Number of fixed and polymorphic synonymous (D_S_, P_S_) and non-synonymous (D_N_, P_N_) substitutions for homologous sites of OG1907 and OG3159 between Sylt and Texel strains.

**Substitution**	**OG1907**	**OG3159**	**% difference**
D_N_	11	2	−81%
D_S_	8	2	−75%
P_N_	150	69	−54%
P_S_	83	197	+137%
Sum	252	270	
DoS = D_n__/_(D_n_ + D_s_)−P_n_/(P_n_ + P_s_)	−0.06	0.24	

*% difference shows the proportional change of each class of substitution between both genes.*

**FIGURE 4:**
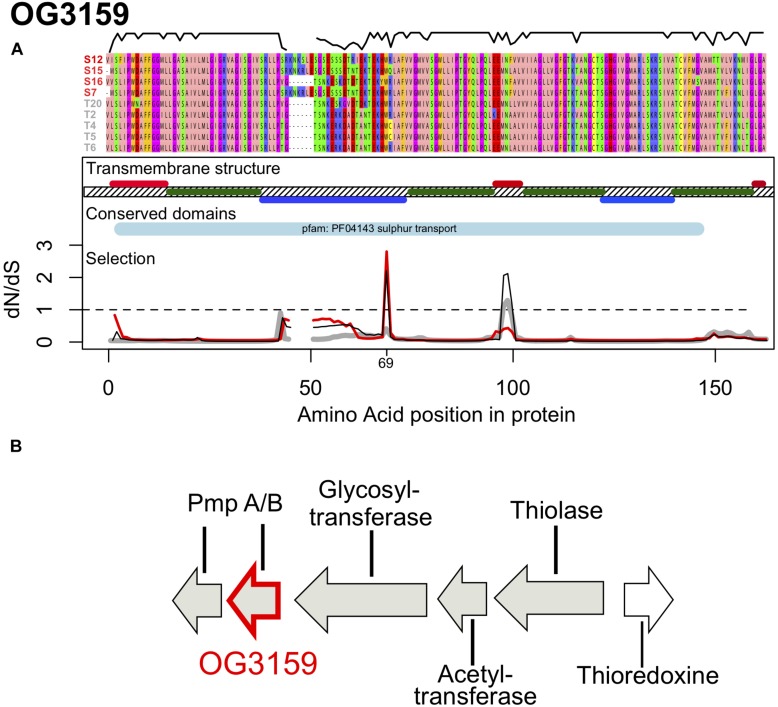
**(A)** Protein alignment, transmembrane structure, conserved domains and selection along the protein sequence of OG3159. The alignment was color coded according to the Zappo color scheme according to their physicochemical properties. The black line above the alignment displays sequence similarity within the alignment. Transmembrane regions are shown in dark green, with periplasmatic regions above the hashed membrane in red and cytoplasmatic regions are shown below in blue. Synonymous to non-synonymous substitution rates (dN/dS) along the sequence are shown for Sylt sequences (red), Texel sequences (gray) and all sequences (Sylt + Texel, black), thus showing contributions of within vs. between location responses to selection. The dashed line shows neutral evolution while peaks above this line indicate positive divergent selection. **(B)** Genomic context of OG3159 showing its genetic neighborhood supporting its role as a sulfur transporter.

The second orthologous group, OG3159 encodes a 153–159 amino acid long protein with a theoretical molecular mass of 15.8 kDa and isoelectric point of 10.2. This protein has four transmembrane domains (positions 15–37; 75–99; 102–121; 141–157) and is predicted to be localized in the cytoplasmic membrane (inner membrane) (Figure 4A). Proteins from this family showed significant sequence similarity with proteins from the YeeE/YeeD family of membrane transporters and especially with a transporter of sulfur-containing compounds, PmpA, identified in *Serratia* sp. ATCC39006 (Gristwood et al., 2011). This conserved domain (PF04143, *e* = 6.9e^–10^) was found in the sequences of all strains used here and the gene was localized in close proximity of a glycosyltransferase, an acetyl transferase, a thiolase and a thioredoxine (Figure 4B), with the latter two suggesting that this protein family is a conserved transporter of sulfur containing molecules.

Large sections of the gene were highly conserved and especially the transmembrane domains of the protein show only little variation between the strains used here (Figure 4A). Consequently, we detected a low dN/dS = 0.145 for the whole gene that was mainly driven by purifying selection on these domains (Figure 4A). In contrast the cyto- and peri-plasmatic domains outside the membrane showed substantially more genetic variation, which also included a 6 amino acid insertion that was found in three out of four Sylt strains (Figure 4A). Accordingly, we found the lowest similarities between locations (79 ± 3.9%) while similarities were significantly higher within Texel (89 ± 7.4%, *t* = 4.350, *p* < 0.001). Similarities within Texel strains were also marginally higher than within Sylt (82 ± 6.5%, *t* = 2.241, *p* = 0.078), while we could not observe significant differences between within Sylt diversity and between Sylt-Texel diversity (*t* = 1.133, *p* = 0.497). Consequently, the distinct distribution of genetic diversity between locations for OG1907 and OG3159 suggests that both genes evolve along different evolutionary trajectories. This difference was also reflected in patterns of synonymous and non-synonymous substitutions observed between locations, that resulted in a DoS = 0.241 for OG3159 suggesting that differentiation might be a result of selection (Table 2). The difference in DoS values was mainly caused by an accumulation of polymorphic synonymous substitutions in OG3159. While both genes had a similar total number of substitutions, fixed and polymorphic non-synonymous (D_N_, P_N_) and fixed synonymous (D_S_) accumulated at a much lower rate in OG3159 than in OG1907. For polymorphic synonymous substitutions (P_S_) this pattern was reversed with more than twice as many substitutions in OG3159 further suggesting that purifying selection removed non-synonymous substitutions.

Despite of purifying selection acting on the majority of protein domains, signatures of positive selection could be detected in the first cytoplasmatic and the second periplasmatic domain (Figure 4A). Position 69 in the first cytoplasmatic domain showed positive selection mainly within the Sylt strains (dN/dS = 2.77, Figure 4A, red line). Adaptive evolution between locations might have been driven by positions 98 and 99, which showed elevated between location selection with dN/dS ratios of 2.064 and 2.120 for the whole data set containing all sequences (Figure 4A, black line). Constraining the data set to sequences from one location revealed purifying selection within the Sylt sequences (dN/dS = 0.377 and 0.403) and neutral evolution within the Texel sequences (dN/dS = 1.106 and 1.286). This between location selection peak fell into the second periplasmatic domain of the sulfur transporter. Together with the Sylt-specific insertion and the high dN/dS ratio within Sylt sequences in the first cytoplasmic domain this might suggest a potential functional role of these domains in ligand specificity for the transport between the cytoplasm and the periplasm (Figure 4A). The observed patterns of between host selection suggest that adaptive substitutions at a few amino acid sites of the two core-genome proteins were shaped by accentuated local selection pressures mediating G x G interactions between sympatric and allopatric combinations of vibrios and oysters.

### The Two Loci Determine the Specific GxG Interactions Between Oyster Larvae and Sympatric Vibrios

**FIGURE 5:**
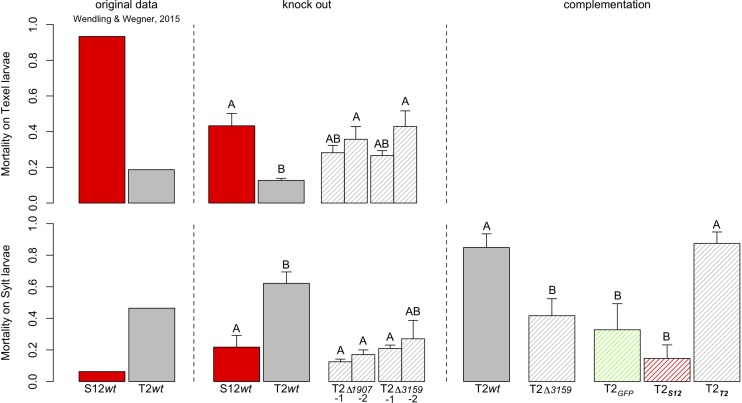
Mortality of larvae exposed to wild-type and mutant strains of the isolates S12 and T2. The left panel shows the original data from Wendling and Wegner (2015). The central panel shows survival after repeated exposure to wild type strains and two knock-out mutants per target gene. Letter codes show pairwise *post hoc* comparisons with groups sharing no letter being significantly different from each other. Eliminating OG1907 from the T2 background removed the host specific virulence patterns by increasing mortality on Texel larvae and significantly decreasing mortality on Sylt larvae. Similarly, the knockout of OG3159 decreased mortality in Sylt larvae, but increased mortality in Texel larvae. The left panel shows the third exposure with the wild type and mutant T2 strains and reciprocal complementation with alleles from either T2 or S12. Letter codes show pairwise *post Hoc* comparisons with groups sharing no letter being significantly different from each other Elimination of T2 significantly reduced mortality compared to the wild type, but the wild type phenotype could fully be restored when the original T2 allele was used for complementation. In contrast, mortality was even further reduced when the mutant was complemented with the S12 allele. Complementation with GFP as procedural control did not change the mortality compared to the knock out mutant.

The role of OG1907 and OG3159 for host specific virulence was assessed by gene knock out. Among the nine isolates, we were able to genetically manipulate only the T2 strain using our genetic tools. The genes OG3159 and OG1907 were deleted and two independent clones (T2Δ3159-1 and 2, T2Δ1907-1 and 2), which were then selected for experimental infection of larvae. We found a highly significant interaction between host sources (i.e., Texel vs. Sylt larvae) and wild type *Vibrio* strains (Deviance Host x *Vibrio* = 25.535, d.f. = 1,8, *p* < 0.001) that qualitatively reproduced our previous results (Wendling and Wegner, 2015; Figure 5). In detail, S12 wild type (wt) induced higher mortality on Texel larvae than T2 wt (estimate = 1.695 ± 0.436, *z* = 3.883, *p* < 0.01), while T2 wt induced higher mortality on Sylt larvae (estimate = 1.547 ± 0.504, *z* = 3.070, *p* = 0.086, Figure 5). Deletion of either gene in strain T2 eliminated this Host × *Vibrio* interaction effect (Deviance Host x *Vibrio* = 1.396, d.f. = 3,16, *p* = 0.707) showing that the host specific virulence patterns were lost. In detail, virulence was significantly decreased on Sylt larvae for T2Δ1907-1 and 2, while it increased mortality on Texel larvae, albeit not significantly (Figure 5). Knock-out of OG3159 showed the opposite pattern as T2Δ3159-1 and 2 lead to decreased mortality in Sylt larvae, but increased mortality in Texel larvae. When the OG3159 gene (OG3159_T__2_ and OG3159_S__12_ for allele T2 or S12 respectively) was constitutively expressed in *trans* from a plasmid in the mutant T2Δ3159, the T2 wt, phenotype was restored only when complementing with the original T2 allele, while virulence was further reduced when the S12 allele was complemented (Figure 5). Altogether our data showed that both OG3159 and OG1907 drive the GxG interactions and are thus necessary for host specific virulence patterns while complementation with alleles from both locations could recreate the respective wild type phenotypes of OG3159.

## Discussion

The large genetic (Thompson et al., 2005) and ecological (Takemura et al., 2014) diversity of vibrios complicates the discovery of genetic factors causing adaptation to different environments or lifestyles. Identification of such genetic factors is particularly relevant for pathogenic vibrios that cause substantial damage to bivalve aquaculture (Paillard et al., 2004; Romalde et al., 2014; Travers et al., 2015; Dubert et al., 2017). However, aquaculture drastically changes the ecological conditions affecting the epidemiology of disease (Pulkkinen et al., 2010; Sundberg et al., 2016), and consequently most virulence factors associated with mortalities in aquaculture have missed the genetic targets of coevolutionary interactions that might be present in wild populations so far. Here, we show that exploiting such coevolutionary interactions between distinct host populations of the Pacific oyster and their sympatric and allopatric vibrios can reveal previously unrecognized virulence factors that mediate host specificity toward genetically distinct populations of Pacific oysters *C. gigas*. We identified two genes (OG1907 and OG3159) that regulate genotype by genotype (GxG) interactions by causing opposite virulence patterns on sympatric and allopatric oyster hosts. Both genes were not classical virulence gene candidates, such as toxin or colonization factors, and only our uninformed approach of filtering out candidates from the whole core genome in combination with genetic knock-out and complementation allowed us to identify these novel genetic targets of local adaptation and coevolutionary interactions between oysters and their *Vibrio* colonizers.

### The Functional Role of OG3159 and OG1907 in *Vibrio*-Oyster Interactions

Annotation of OG3159 revealed a Yee/YeD domain present in all sequences analyzed here. This domain was originally characterized in *Serratia* sp. (strain ATCC 39006), where it is involved in the production of a pigment antibiotic prodigiosin as part of a four gene operon with PigS and three membrane bound molecules PmpABC functioning as a transporter for sulfur molecules (Gristwood et al., 2011). While the PigS and PmpC were absent from the cluster in the *Vibrio* sequences analyzed here, the proximity of genes catalyzing reactions containing sulfur compounds (Thioredoxine, Thiolase, Figure 4) suggests that this cluster is involved in transport of sulfur containing components. This putative function together with the localization in the inner cytoplasmatic membrane, that is not in direct contact with the oyster host, does not intuitively explain how this gene is involved in host-population specific virulence. Our genetic analyses do, however, clearly demonstrate its role in host specificity, because the complementation with the location specific alleles (OG3159_S__12_ and OG3159_T__2_) was sufficient to switch between the wild type virulence phenotypes of T2 and S12 (Figure 5). Complementation T2Δ3159 with the respective alleles of T2 and S12 mainly changed the protein sequences of the first cytoplasmatic and the second periplasmatic domain (Figure 4A). More amino acid substitutions could be observed between the S12 and T2 alleles in the first cytoplasmatic domain than in the second periplasmatic domain. Yet, substitutions in the cytoplasmatic domain did not show any signature of positive selection (with the exception of position 69, which codes for the same amino acid in both alleles, Figure 4A). The second periplasmatic domain, however, matches the between location peak of selection (Figure 4A), and complementation leads to an amino acid change from Alanine in T2 to Phenylalanine in S12. Since complementation exchanged the whole allele and not just the second periplasmatic domain, we cannot say for certain that this specific amino change causes the phenotype switch. Nevertheless, over all alleles substitution patterns suggest that selection was favoring the different amino acid in Sylt and Texel strains by changing the predominant Leucine at position 99 in four out of five Texel strains to a Phenylalanine in three out of four Sylt strains (Figure 4A), thus changing from aliphatic to aromatic properties that might increase binding to hydrophobic substrates (Betts and Russel, 2003) and thus target different organosulphur substrates. Organosulphur compounds like prodigiosin, the target of the OG3159 homolog PmpB in Serratia (Gristwood et al., 2011), have been shown to have antimicrobial properties (Lapenda et al., 2015). They have also been shown to interfere with signal transduction pathways and act as an immunodepressive (Williamson et al., 2006). So, if different OG3159 alleles do indeed show different affinities for different organosulphur compounds for the production of different prodigiosin-like molecules it could mediate competitivity/resistance or the interaction with the oyster host even though the protein itself is not in contact with the host or other bacteria colonizing the oyster.

In the case of OG1907, the presence of a signal peptide and localization outside of the cell indicates that this gene is an extracellular protein that could potentially mediate the interaction with the oyster host directly. However, functional homology of conserved domains provides only little information on how such an interaction might look like. The only conserved domain was a Glycosyl hydrolase 3 domain, which was only predicted for the strains isolated on Sylt but not for strains isolated on Texel. Furthermore, even for the Sylt strains the confidence for the glycosyl hydrolase domain prediction was comparatively low (*e* = 6.4e−04) casting doubt on a function in saccharide modification of this gene. More interestingly, the gene was located directly upstream of the *cusABC* cluster in all strains. *cusABC* forms a periplasmatic copper efflux pump (Chacon et al., 2014) and has been implicated in copper homeostasis and resistance in various gram negative bacteria including vibrios (Hernandez-Montes et al., 2012). Moreover, also *copA*, which encodes a copper-exporting P-type ATPase, has been previously demonstrated to be an essential virulence factor in *V. tasmaniensis* (strain LGP32; Vanhove et al., 2016) and *V. crassostreae* (J2-9; Bruto et al., 2017). In LGP32 *copA* has been further shown to be required for copper resistance, intracellular survival and cytotoxicity in phagocytes (Vanhove et al., 2016). Since oyster use copper for oxygen transport, copper concentrations in oysters can be very high (Amiard et al., 2008). Together with the results found in *V. tasmaniensis* (Vanhove et al., 2016) our findings might suggest a more general role for copper resistance in oyster associated life styles of vibrios. In addition, copper concentration in Sylt shellfish (blue mussel, *Mytilus edulis*) increased twofold between 1997 and 2004 reaching up to four times higher levels than the water background while concentrations on Texel stayed constant with twice the concentration as found in open water (Essink et al., 2005). The different accumulations of copper in shellfish might thus have formed the selective background driving the differentiation of the gene cluster containing OG1907 between Sylt and Texel that we observed here. We are aware that predicting the detailed function of genes from homology to known proteins will always harbor a speculative component. This also applies to the functional context suggested above, and further experiments are needed to gain a deeper insight into the functional relation of these genes to the host specific virulence phenotypes.

### Selection Pressures

The different and partly opposing patterns of sequence diversity and nucleotide substitutions found for OG1907 and OG3159 indicate that different evolutionary forces and selection pressures shaped diversity of both genes. Yet, the gene knock-outs of both genes caused the specific virulence patterns against sympatric and allopatric oyster hosts of the wild type strains to break down. This obvious link to the host-specific virulence phenotypes may suggest that sites that display positive selection between locations might represent the mutations that were selected to evoke host specific virulence patterns. To fully understand what is driving the evolution of host-specific virulence patterns, one must also understand the selective pressures that cause adaptation. Increased virulence is often associated with increased fitness (Johnson, 2013), leading to the prediction that sympatric vibrios should cause higher mortality. Here, we find the opposite patterns that vibrios express lower virulence on their sympatric oyster hosts. Lower virulence on sympatric strains was not just observed in the 9 strains sequenced here but in the majority of all strains isolated on Sylt and Texel (*i.e*., 61%, Wendling and Wegner, 2015) indicating that the evolution of host specific virulence is rather a rule than an exception. Since mortality in disease always reflects the balance between resistance/tolerance of the host and virulence of the pathogen (Feis et al., 2016), two adaptive scenarios could explain this pattern: For one, oysters could have evolved resistance against their local vibrios and use the OG1907 protein or the OG3159 ligands as molecular targets for recognition and resistance evolution. The dominant inheritance found in larvae of F1 crosses between oysters from both locations supports this as it signifies a strong host genetic component (Wendling and Wegner, 2015). Furthermore, it was already shown that cellular immune activity was stronger against sympatric vibrios in adult oysters from the same locations (Wendling and Wegner, 2015), suggesting specific immune stimulation in sympatric combinations of vibrios and oysters. While it is unclear to which stage the immune system of larval oysters is developed (Zhang et al., 2012) any increase in resistance or tolerance against infection can be considered adaptive for the oyster host and will be selected for.

On the other hand, the position specific patterns of positive selection found between locations for both *Vibrio* genes shows that also the *Vibrio* strains themselves responded to selection by adaptation. If the *Vibrio* strains used here indeed adapted to their respective location, higher virulence would not correlate to higher fitness. Indeed, maximizing virulence might not always be adaptive when transmission is impeded by reduced host density [i.e., virulence transmission trade off (Anderson and May, 1982)]. Aquaculture can select for higher virulence (Sundberg et al., 2016) also because host densities are kept high by artificial supplies of new hosts. Such a transition toward higher virulence was already observed in an aquaculture background when plasmid acquisition turned a benign oyster colonizer into an emerging pathogen (Bruto et al., 2017). Therefore, the natural oyster reefs on Sylt and Texel, where densities naturally fluctuate (Troost, 2010; Reise et al., 2017), might actually select for lower virulence, that was repeatedly observed as a result of host-parasite coevolution (Acevedo et al., 2019). This could mean that *Vibrio* strains get selected that colonize oysters with causing only little harm and become part of the natural oyster microbiome. In general, the high abundance of vibrios in the hemolymph of healthy oysters shows that vibrios are enriched in oysters compared to the surrounding sea water and are important components of the hemolymph microflora (Lokmer et al., 2016). Some strains are even considered to be beneficial and were suggested as probiotic candidates (Gatesoupe, 1999) further suggesting that colonizing the oyster host does not necessarily lead to the evolution of virulence but can also select for mutualistic interactions. Detailed experiments investigating the colonization process against the backdrop of oyster resistance are, however, needed to differentiate between the alternative scenarios.

## Conclusion

By exploiting coevolutionary *Vibrio*-oyster interactions in natural oyster beds we were able to identify two genes that could explain the host specific virulence patterns. These genes are putatively involved in functions very different from previously described virulence genes and would have never been picked as target genes to explain variation in virulence. Yet, our genetic analyses, although it is based on a limited number of strains, can demonstrate their functional role in the contrasted virulence patterns between sympatric and allopatric oyster hosts, highlighting the use of our approach to explore the “dark matter” of virulence genes. While virulence toward oysters can be an ancestral trait in the *Splendidus* group (Bruto et al., 2018), many other virulence factors have been discovered (Hasegawa et al., 2008; Labreuche et al., 2010; Duperthuy et al., 2011; Goudenege et al., 2014; Lemire et al., 2014). This diverse arsenal of population specific weapons does not only reflect the overall genomic and ecological diversity of vibrios (Thompson et al., 2005; Takemura et al., 2014), but also the diversifying forces inherent to host-parasite coevolution. The discovery of the two genetic factors in this study not only adds to this diversity, but describes genes that are responsible for host specific virulence patterns that constitute local adaptation and/or function as targets for coevolutionary interactions. Since only a few mutations in both genes showed signs of positive selection between locations, evolution of specificity might proceed swiftly and the increasing number of mass mortalities caused by disease also in natural populations (Fey et al., 2015) calls for a mechanistic understanding of the underlying causes. The analysis of oyster-*Vibrio* interactions from natural oyster beds that are largely independent from alteration of disease dynamics often caused by aquaculture influences (Pulkkinen et al., 2010; Sundberg et al., 2016) thus offers a promising experimental system to investigate disease dynamics and selective pressures in the wild (Le Roux et al., 2016).

## Data Availability

Assembled sequences are available from NCBI under the accession numbers: VFEB00000000 (T20); VFEC00000000 (T6); VFED00000000 (T5); VFEE00000000 (T4); VFEF00000000 (T2); VFEG00000000 (S7); VFEH00000000 (S15); VFEI00000000 (S12); VFEJ00000000 (S16).

## Author Contributions

KW conceptualized the study. UJ sequenced the strains. ZM performed the initial assemblies and annotations. KW and MB performed the bioinformatics analyses. DP and FLR knocked out and complemented the target genes. MA-B, BP, KW, and FR bred oysters and performed the infection experiments. KW, MB, and FR wrote the manuscript. All authors commented and approved the final version of the manuscript.

## Conflict of Interest Statement

The authors declare that the research was conducted in the absence of any commercial or financial relationships that could be construed as a potential conflict of interest.

## Supplementary Material

TABLE S1 Primers used in the present study.

TABLE S2 Strains and plasmids used or constructed in the present study.
